# Upgrade of the SPECIES beamline at the MAX IV Laboratory

**DOI:** 10.1107/S1600577521000564

**Published:** 2021-02-05

**Authors:** Esko Kokkonen, Felipe Lopes da Silva, Mikko-Heikki Mikkelã, Niclas Johansson, Shih-Wen Huang, Jenn-Min Lee, Margit Andersson, Antonio Bartalesi, Benjamin N. Reinecke, Karsten Handrup, Hamed Tarawneh, Rami Sankari, Jan Knudsen, Joachim Schnadt, Conny Såthe, Samuli Urpelainen

**Affiliations:** a MAX IV Laboratory, Lund University, Box 118, 221 00 Lund, Sweden; b Nano and Molecular Systems Research Unit, University of Oulu, Box 3000, 90014 Oulu, Finland; c Environmental and Chemical Engineering, University of Oulu, Box 4300, 90014 Oulu, Finland; e Department of Physics, Tampere University, PO Box 692, 33101 Tampere, Finland; d Division of Synchrotron Radiation Research, Department of Physics, Lund University, Box 118, 221 00 Lund, Sweden

**Keywords:** APXPS, RIXS, beamlines, MAX IV

## Abstract

The SPECIES beamline has been transferred to the new 1.5 GeV storage ring at the MAX IV Laboratory. Several improvements have been made to the beamline and its endstations during the transfer. Together the Ambient Pressure X-ray Photoelectron Spectroscopy and Resonant Inelastic X-ray Scattering endstations are capable of conducting photoelectron spectroscopy in elevated pressure regimes with enhanced time-resolution and flux and X-ray scattering experiments with improved resolution and flux. Both endstations offer a unique capability for experiments at low photon energies in the vacuum ultraviolet and soft X-ray range. In this paper, the upgrades on the endstations and current performance of the beamline are reported.

## Abstract 2

The transfer and upgrade of the SPECIES beamline and its endstation to the 1.5 GeV storage ring at the MAX IV Laboratory is reported.

## 1. Introduction   

The SPECIES beamline is a soft X-ray undulator beamline on the 1.5 GeV storage ring at MAX IV Laboratory in Lund, Sweden. The beamline covers the photon energy range from 30 to 1500 eV with variable polarization. The X-rays are generated using an elliptically polarizing undulator, EPU61 (Wallén *et al.*, 2014 ▸), and monochromated with a plane-grating monochromator illuminated with collimated light [cPGM (Follath *et al.*, 1998 ▸)]. The beamline was originally built on the MAX II storage ring, which was decommissioned at the end of 2015. The entire beamline and the endstations were then transferred to the new MAX IV facility where user operation began in 2019. The exact details of the beamline are mostly unchanged from the previous configuration in 2015 (Urpelainen *et al.*, 2017 ▸), and only the upgrades and changes are discussed in this paper.

**Figure 1:**
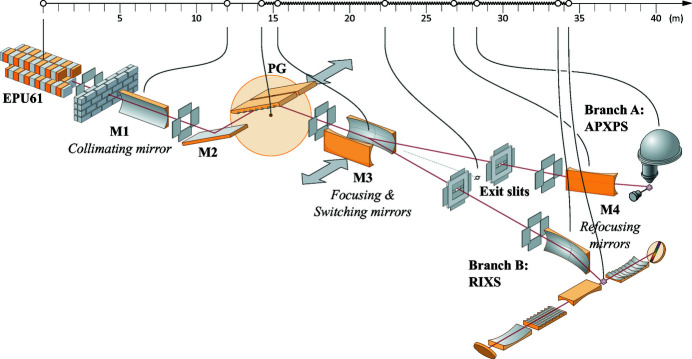
Layout of the SPECIES beamline showing the most important optical components. The first mirror (M1) collimates the beam vertically, with focusing done using the third mirrors (M3) (only vertically for APXPS and vertically and horizontally for RIXS). Refocusing mirrors (M4) are used to focus on the optimum spot for the samples.

The beamline offers two branches: branch A is dedicated to ambient pressure X-ray photoelectron spectroscopy (APXPS) and branch B to resonant inelastic X-ray scattering (RIXS). The main technique in the APXPS branch is X-ray photoelectron spectroscopy (XPS), but it has also capabilities for X-ray absorption spectroscopy (XAS) and near-nedge X-ray absorption fine-structure (NEXAFS) experiments in total or partial electron yield mode. The RIXS branch can also perform XAS and NEXAFS measurements by recording the emitted electrons or photons. While the APXPS endstation is also capable of measurements in the UV range and thus qualifies as an ambient-pressure UV photoelectron spectroscopy (APUPS) instrument, we will only refer to it as the APXPS endstation throughout this paper. A schematic layout of the beamline is presented in Fig. 1 ▸ including distances between optical elements.

In the surface science community, ultrahigh vacuum (UHV) XPS is a well known and trusted method for obtaining detailed information on the electronic structure of surfaces as well as elemental and chemical composition (Hüfner, 2013 ▸). While exposing surfaces to gases is also possible in UHV systems, higher pressures are normally inaccessible. In contrast, APXPS makes it possible to study materials and their properties under conditions that more closely mimic those occurring under real-world processes and phenomena (Bluhm *et al.*, 2007 ▸; Ogletree *et al.*, 2009 ▸; Schnadt *et al.*, 2020 ▸). Experiments at ambient pressure (in the context of this paper, ambient pressure refers to pressures of ∼1 mbar) pose a serious challenge, however, as the electron mean free path in gas is of the order of millimetres (Ogletree *et al.*, 2002 ▸). The method employed at SPECIES is to use the ‘Lund cell’ approach (Knudsen *et al.*, 2016 ▸), which uses the cell–incell concept (Schnadt *et al.*, 2012 ▸; Tao, 2012 ▸; Starr *et al.*, 2013 ▸). Here, the sample environment is created inside an ambient pressure (AP) cell, which itself is placed inside a UHV vacuum chamber. The concept enables changing the sample environment swiftly from UHV conditions to ambient pressure since the AP cell can be removed in-vacuum. Another advantage is the possibility for fast exchange of gases due to the small volume of the cell. Typically in APXPS setups, an aperture of the electron analyser is placed very close to the sample surface in order to minimize the distance the electrons have to travel in the high-pressure region, thereby increasing the transmission of electrons.

The SPECIES beamline offers the possibility of conducting APXPS measurements using low photon energies which make possible avenues of research that might have been previously neglected. In particular, the ability to measure valence band spectra in elevated pressure regimes is interesting for correlating different phenomena on the surface such as the absorption of various molecules, which might not give very strong signal on the core-levels. However, the use of low photon energies (<100 eV) will ultimately result in low kinetic energy photoelectrons as well. As was recently reported by Held *et al.*, the transmission of low kinetic energy electrons through a layer of high pressure gas can be very low (Held *et al.*, 2020 ▸). Thus, one may have to take into account the consequence that high pressure has on the electron transmission and make compromises. At SPECIES, the very high flux in low photon energies is an advantage that will help even in situations where transmission might be otherwise low.

The APXPS setup has been previously described (Schnadt *et al.*, 2012 ▸; Knudsen *et al.*, 2016 ▸; Urpelainen *et al.*, 2017 ▸) and here we will only give the details of the upgrades that have been carried out on the system in connection to the transfer to the new facility. The paper is organized as follows: details of the upgrade of the APXPS endstation, details of the RIXS endstation, the performance of the beamline on the new 1.5 GeV storage ring, and examples of research using the APXPS endstation.

## 2. Upgrade of the APXPS endstation   

**Figure 2:**
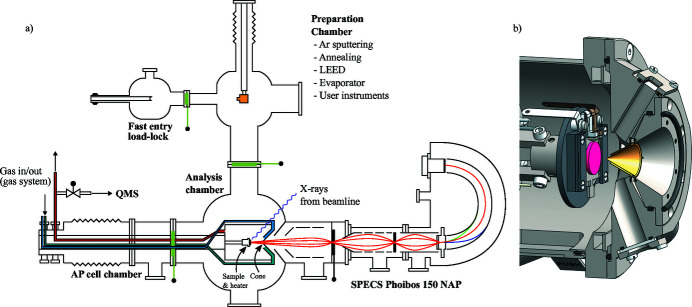
(*a*) Schematic layout of the APXPS endstation showing the different chambers. During measurements the cell is located inside the analysis chamber where the synchrotron beam enters it. Samples are transferred from the load-lock through the preparation chamber (above the analysis chamber) and into the cell using the UHV manipulator inside the preparation chamber and a wobble stick in the analysis chamber. (*b*) Close-up view of the drawing of the AP cell and the sample inside it showing the real dimensions. Note that half of the the front piece of the cell is cut in this view to better see the sample and the double-cone.

The APXPS endstation is a surface science instrument equipped with a SPECS Phoibos 150 NAP electron energy analyser. The layout of the endstation is shown in Fig. 2 ▸. The endstation has two manipulators for sample movement. The UHV manipulator is intended for measurements without the AP cell, in UHV conditions. The UHV manipulator can be used for in-vacuum sample preparation and characterization inside the preparation chamber, which is situated above the analysis chamber. The preparation chamber has permanent instruments for Ar^+^ sputtering, sample heating, low-electron energy diffraction (LEED) characterization of sample surfaces, and for dosing gases up to pressures of ∼10^−5^ mbar. In addition, the preparation chamber has ports available for infrequently used equipment, such as evaporation sources, or user equipment. The additional ports are behind gate valves, allowing equipment installation without the need for venting the whole preparation chamber.

The AP cells are installed on another manipulator, placed horizontally and facing the analyser. This placement makes it possible to keep the AP cell and its manipulator isolated from the vacuum of the analysis chamber, thus making maintenance, repairs and bake-outs faster and more accessible since only a smaller chamber is vented. For measurements, the AP cell has to be docked onto the analyser using its own manipulator. Once docked to the analyser, it is locked in place with a bayonet mechanism, which keeps the cell in place but allows sample movement. Proper sample movement is important in order to characterize several areas on samples and for mitigating X-ray-induced beam damage. Samples are transferred from the analysis chamber into the AP cell with a transfer wobblestick, which is also used to operate the door that seals the volume within the cell and keeps high-vacuum conditions outside it. The sample holders have the typical SPECS/Omicron flag-type shape with a modified thermocouple design.

All AP cells have two gas inlet lines. Both lines can be connected to a dedicated gas system, where several gases can be installed simultaneously. The flows from each gas line are independently controlled using mass flow controllers. This allows mixing and accurate control of the gas composition, which is fed into the cell. Vapours from liquid sources can be fed into the cell using, for example, leak valves. Special sources can also be installed (for example in the case of the atomic layer deposition cell, see Section 2.3).

During the installation and commissioning phase of the beamline at the new MAX IV 1.5 GeV storage ring, several improvements were made on the APXPS endstation. The electron spectrometer is a commercial system purchased from SPECS Surface Nano Analysis GmbH, Berlin, Germany. The NAP 150 spectrometer houses a differentially pumped electrostatic lens system allowing for ambient pressure measurements while still keeping the detector and hemisphere at high vacuum. The spectrometer was originally equipped with a CCD camera-based electron detection system. As part of the beamline transfer, this detector system was replaced with a faster acquisition scheme involving microchannel plates (MCPs) and a delay-line detector (DLD). The detector is a 3D-DLD4040-150 from Surface Concept GmbH and it consists of MCPs in a chevron stack and two layers of delay lines (*X* and *Y*) in a meander structure. The electron signals from the delay line are analysed by the readout electronics including a constant fraction discriminator (CFD) for pulse shaping and a fast time-to-digital converter (TDC) for time stamping. All detector electronics are housed in a single rack-mounted electronics box. The active area of the detector is approximately 40 mm in diameter, which is converted by the electronics into an image with a size of about 800 × 1000 pixels. The binning of the detector image can be changed in the software to reduce the size of the saved image if necessary. The detector is capable of reaching a count rate in the MHz range before saturation is reached.

The DLD system allows synchronization of the detector with any other source of external pulsing, or gating it to inhibit electron detection. As an example of this, we have recently demonstrated that it is possible to trigger the detector at the same time as pneumatically actuated valves in order to achieve pulses of reactant gas. Such synchronization is essential for precise gas control as well as data acquisition, which is required for accurate alignment of gas pulses temporally to each other while also providing a convenient platform to program advanced experiment automation (Redekop *et al.*, 2020 ▸).

The differentially pumped lens system of the analyser has also been upgraded with a new version of the SPECS pre-lens (Release 3). An important feature of the pre-lens is that it should create a leak-tight connection with the AP cell. The new pre-lens features special guiding elements that ensure that the AP cell is always placed to the same position with respect to the pre-lens. During the upgrade, the pre-lens electronics were replaced with newer, modernized versions. The Release 3 version of the pre-lens offers approximately one order of magnitude higher transmission with a similar resolution setting as the Release 2 version of the pre-lens system (SPECS, 2020 ▸).

Further developments of the endstation to enhance its operation are ongoing. The gas mixing system will consist of gas panels with independent gas lines for most common gases, such as O_2_, CO, CO_2_, H_2_, N_2_ and Ar. Each gas line will include mass flow controllers and pumping capabilities for easy exchange of gas bottles. For gases that need them, there are also gas purifiers and the capability of using condensers to remove impurities such as water. Independent gas lines will also reduce the potential for cross contamination. The panels will be incorporated into the MAX IV gas standard and control logic which allows for a simple, remotely operated system.

A second load-lock chamber has been designed, which will be installed next to the vacuum chamber where the AP cell typically is located. The idea of the second load-lock is to have a small volume that is detachable from the load-lock itself that can be taken, for instance, into a glove-box filled with an inert gas for inserting samples that are sensitive to air. This so-called controlled atmosphere load-lock allows the user to have a well controlled sample transfer atmosphere from the sample loading to the measurement without having to expose the sample to air or vacuum.

Several ambient pressure cells have been constructed for use at the APXPS endstation. These include one general-purpose cell (standard cell) for most common measurements that do not require any special conditions, one cell dedicated to measurements with corrosive gases (sulfur cell) such as sulfur-containing gases, and one cell dedicated for atomic layer deposition research (ALD cell). The important parameters in the present cells are tabulated in Table 1 ▸. All cells have the same volume of about 200 ml, excluding the gas tubes.

**Figure 3:**
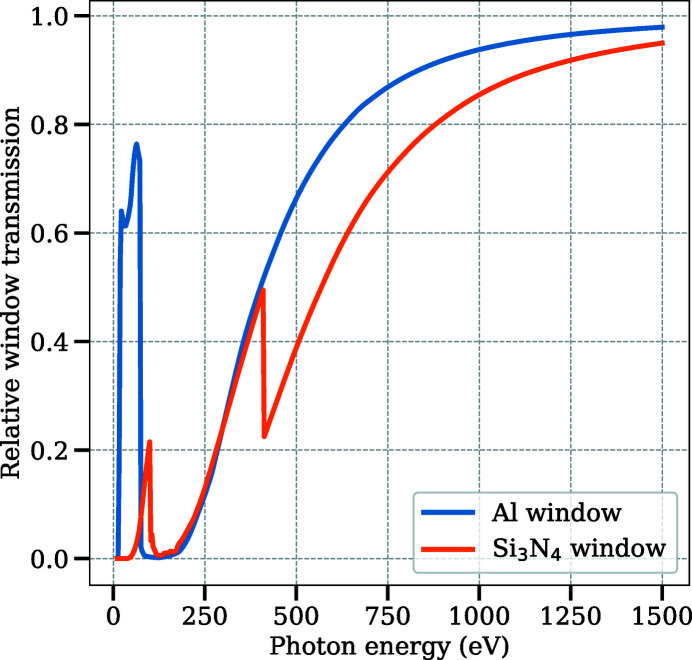
Transmission of synchrotron radiation through 200 nm of Al or Si_3_N_4_ calculated using the CXRO database (Henke *et al.*, 1993 ▸). The Al window has a good transmission up to the Al 2*p* edge at around 72 eV and begins to increase again after 200 eV. The Si_3_N_4_ window has much smaller transmission due to the Si 2*p* and N 1*s* edges at about 100 and 400 eV. Both windows have relatively good transmission above 500 eV.

All cells share the same window design for the incoming synchrotron radiation. Different windows are available depending on the requirements of the experiment. Currently, there are windows with 200 nm thickness of Si_3_N_4_ with a thin, protective Al coating (from Silson Ltd, UK) or pure Al (from Luxel Corporation, USA). The calculated transmission curves for these materials differ substantially in the low-energy range as can be seen in Fig. 3 ▸. Calculations are performed using the Center for X-ray Optics (CXRO) database (Henke *et al.*, 1993 ▸).

The cone through which electrons enter the analyser is identical in each cell. The size of the cone aperture ultimately defines the maximum achievable pressure of the cell. With a 0.3 mm diameter the maximum pressure is about 20 mbar, while with the other cones available at the beamline (0.5 mm and 1.0 mm diameters) the maximum pressures are lower. Consequently, larger cone apertures will yield higher electron transmission in situations where the footprint of the beam is large (for example when using an X-ray anode as a light source). The accuracy of the sample positioning in front of the aperture is also dependent on its diameter. With smaller cone aperture, the sample has to be placed closer to the cone which yields stricter requirements for the alignment of the sample with respect to the analyser and synchrotron beam. A rule-of-thumb is to place the sample approximately twice the aperture diameter away from the cone aperture. At this distance, the pressure on the sample surface will remain approximately homogeneous despite the pumping effect through the cone (Bluhm *et al.*, 2007 ▸). This distance is often visually confirmed using a camera which looks at the cone and the sample surface.

### 2.1. Standard cell   

**Figure 4:**
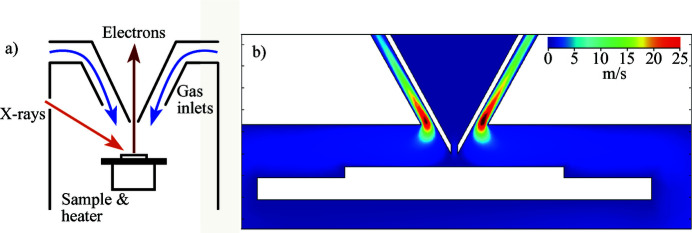
(*a*) Schematic view of the cell with the double-cone system for gas inlet. (*b*) Results of the gas flow simulations in a simplified model cell. In this case the results show the flow with an inlet pressure of 1 mbar. The colour scale shows the velocity magnitude of the gas in units of m s^−1^. The simulation shows that the gas passes over the sample surface in a uniform manner.

The standard cell (designed in collaboration with Synchrotron SOLEIL), as well as the other cells, follow the basic design principles of the original cell from SPECS (Schnadt *et al.*, 2012 ▸). Some improvements have been made, however. The most notable change is the method of releasing the gas into the cell. Gas is introduced to the cell using the so-called double-cone inlet system, where the gas inlets are directed towards the sample surface from the same direction as the analyser. The double-cone inlet system is schematically shown in Fig. 4 ▸(*a*). The cone which separates the cell vacuum from the pre-lens vacuum has another, larger, cone around it and the gas enters the cell volume from there. Since the cone(s) are often very close to the sample surface, this ensures that the response from the surface is faster than if the gas was introduced at the back of the cell, as was done in the original AP cell.

Gas flow simulations have been carried out using this design, with an example shown in Fig. 4 ▸(*b*). The simulations were performed using the *Molecular Flow* module in the *COMSOL Multiphysics* software (COMSOL, 2020 ▸). The results indicate an efficient flow of gas towards the spot where the X-ray beam hits the sample surface, and that entire sample is reached by the flow uniformly. With the double-cone system, it is very unlikely that the gas bypasses the sample surface altogether, a scenario that could trouble setups where the gas inlet is located elsewhere. The endstation is equipped with a quadrupole mass spectrometer (QMS), which can probe gas composition in the outlet and one of the inlet lines. Since the double-cone system directs the gas flow towards the sample surface, reactivity measurements using the QMS should be more detailed, than in a geometry where the gas inlet is behind the sample.

The cell is equipped with a miniaturized Pirani gauge (MicroPirani model #905 by MKS Instruments) located on one of the ports facing the sample surface. The MicroPirani is capable of measuring the pressure range from 10^−5^ to 1000 mbar and gives the possibility of measuring pressures very near the sample, thereby increasing accuracy. The MicroPirani has been initially tested and is available to users soon.

The sample heating system in the cell was chosen to be based on resistive heating using a button heater (Model #101275 from HeatWave Labs, Inc.). In the button heater, the resistive platinum filament is housed inside an Al_2_O_3_ body. The button heater itself is placed just below the sample holder, where the heat can be conducted and radiated into the sample holder and to the sample itself. According to the specifications the heater can be operated up to 1200°C in an oxygen atmosphere, but we have chosen to limit the highest temperature to approximately 600°C due to the presence of sealing O-rings very close to the heater. As the filament wire and the housing of the button heater itself can be catalytically active (Palomino *et al.*, 2017 ▸), it is often imperative that the QMS data are verified by measuring another reference data set without the sample inside the cell. A possible solution for the issue is to replace the filament with more inactive material, *e.g.* graphite.

The standard cell is also equipped with a cooling channel near the sample stage itself. The purpose of this is to increase the rate of cooling from high temperatures towards room temperature (RT). The same cooling channel can also be used to cool the samples below RT, by flowing a cold gas or liquid through the channel. So far it has been demonstrated that by flowing cold nitrogen gas through the channel it is possible to cool the samples down to −30°C. Cooling the samples further extends the available sample environments and is useful, for instance, in the cases where controlling the relative humidity inside the cell is desired (Lin *et al.*, 2021 ▸). The same cooling could also be used for the investigations of ice on surfaces.

### 2.2. Sulfur cell   

The beamline provides also a cell for corrosive and sticky gases. The principal design is nearly identical to the standard cell. The general idea, however, is to provide a dedicated setup for use with these types of gases. With a dedicated setup, problems caused by cross-contamination with experiments that require clean conditions can be avoided. For this reason, the sulfur cell ideally contains its own set of piping on the inlet and outlet side which would be completely separate from the piping that is shared with the standard and ALD cells.

To facilitate faster transport of gases into the cell and decrease condensation into the tubes, they can be heated in vacuum with resistive heating elements. The heating element is wrapped around the longest section of the tubes in-vacuum, which allows heating them to a temperature of up to 200°C.

The sulfur cell will have its own, independent, gas panel system with individual gas lines and mass flow controllers for specific gases. As sulfur-containing gases are typically very corrosive, the rationale is to avoid cross-contamination with the other cells as much as possible. The gas panel for the sulfur cell will be thus isolated from the gas system of the other cells.

Material choices for the sulfur cell are somewhat restricted due to the highly corrosive nature of the used gases. The material for the sealing O-rings was chosen to be FFKM-type perfluoroelastomer which provides enhanced chemical resistance and better stability at higher temperatures. Additionally, typical K-type thermocouple material is not stable in an atmosphere of sulfur-containing gas. Therefore, a C-type thermocouple material will be used instead.

Allowed sulfur containing gases will depend on a risk analysis that is done on a case-by-case basis. Once the sulfur cell has been fully commissioned, the list of gases will be determined and the information will be available through the beamline website.

### 2.3. ALD cell   

Atomic layer deposition (ALD) is a technique to grow uniform layers of material with high degree of control (Miikkulainen *et al.*, 2013 ▸). A substrate is exposed to pulses of two (or more) precursor gases in a sequential manner, thereby achieving a highly ordered growth of atomic layers. APXPS is a very convenient tool for studying ALD processes since the pressure and temperature ranges needed for optimal growth are within the ranges of the typical AP cells in use at SPECIES. APXPS has been recently demonstrated as a very powerful tool for *in situ* and *operando* investigations into the first half-cycles of the ALD processes (Head *et al.*, 2016 ▸; Timm *et al.*, 2018 ▸; Temperton *et al.*, 2019 ▸; D’Acunto *et al.*, 2020 ▸).

The ALD cell at SPECIES was designed and constructed (collaboration between University of Helsinki and MAX IV) in order to achieve a gas flow that would mimic flows in real ALD reactors. For this purpose, the cell contains two independent gas inlet lines to be used with two different precursor gases. These gas lines are built inside the cell so that they point towards the sample surface, ideally resulting in a laminar-like flow across the surface. Additionally, there is an outlet or pumping line on the other side of the sample also facing the sample surface, further increasing the likelihood of flow with laminar characteristics. In the same way as the other AP cells, the ALD cell has relatively long gas tubes (∼1.5 m) from the feedthrough to the cell itself. The tubes are heated in the same way as in the sulfur cell. Additionally, the cell walls can also be heated using independent heating elements. The aim of these extra heating elements is to facilitate faster transport of precursor gases and to prevent the formation cold spots around the cell chamber.

The outlet line of the ALD cell is connected to the QMS in the same manner as in the standard cell. This enables accurate measurement of the reaction products, which often show response to the ALD reactions happening on the surface due to broken ligands and other fragments that are normal during the specific ALD process in question.

Since the cell walls are heated, it was decided that no cooling lines would be made for the cell. The lack of cooling has some restrictions on the type of O-rings that could be used for sealing various parts of the cell. Therefore, also for the ALD cell, FFKM-type O-rings are used. The walls of the cell and the gas tubes will be very likely coated with different metals as ALD experiments are conducted. So far, this coating has not been seen interfering with the experiments, as long as the pumping efficiency of the cell and the tubes is not affected (*i.e.* as long as there is no accumulation of ‘sticky’ gases in time). The cell is designed with a high degree of exchangeability in mind, allowing easy replacement of contaminated or dirty components.

### 2.4. Control systems   

The control systems for the beamline were already partially developed when the beamline was commissioned and operated on the MAX II storage ring. The control system and their design principles are detailed elsewhere (Lindberg *et al.*, 2015 ▸; Sjöblom *et al.*, 2016 ▸). However, several improvements have been implemented after the move to MAX IV Laboratory especially since the other beamlines in the facility share the same control system logic.

The APXPS endstation has gone through an overhaul of the vacuum control systems. Most vacuum pumps, gauges and valves are connected to the same control system through an automation interface and *Tango Controls* (Tango, 2020 ▸) based logic. This allows for easy control of all vacuum-related components as well as logging of crucial parameters. Moreover, user safety and equipment protection can be increased through many different vacuum pressure setpoints and other monitorable parameters.

The manipulators in the APXPS endstation and the monochromator can be controlled directly from the XPS measurement software (*SpecsLab Prodigy*). This allows sophisticated measurement modes to be made where, for example, sample movement is performed automatically during the electron spectrum acquisition. Additionally, the monochromator can be moved between measurements, making easy and automated partial electron yield measurements possible.

## 3. RIXS endstation   

The Resonant Inelastic X-ray Scattering (RIXS) endstation is designed with a high degree of sample versatility in mind. The experimental endstation consists of a customized vacuum chamber for the spectrometers and a load-lock which allows sample transfer from air or from portable vacuum suitcases. The endstation is equipped with a high-stability manipulator with exchangeable sample rods. Each rod is 60 mm in diameter and 600 mm long and can hold several samples. Different types of sample environments are available, such as helium-cooled, nitrogen-cooled and standard rods. Depending on the cooling type, the sample temperature can reach 10 K (helium cooled) or 80 K (nitrogen cooled). A micro-jet setup and different types of liquid cells are currently being developed to expand the *in situ* measurement capability.

The main chamber of the endstation has additional detectors for recording XAS or NEXAFS spectra. These measurements can be made by measuring the drain current from the sample itself, or by recording the total amount of emitted photons with dedicated detectors such as photodiodes or MCP detectors.

The endstation currently houses two spectrometers both mounted perpendicularly with respect to the incident photon beam and opposite to each other. The first spectrometer is a modified Scienta XES350 (Grace) operated in slitless mode (Nordgren *et al.*, 1989 ▸). The Grace spectrometer houses three spherical gratings with line densities of 300 lines mm^−1^ (3 m radius), 400 lines mm^−1^ (5 m radius) and 1200 lines mm^−1^ (5 m radius). The Grace spectrometer can cover the photon energy range from 50 to 1500 eV at reasonable resolving power (hundreds) by operating it in the first or second diffraction order. Due to the relative low photon flux above 650 eV, experiments at the Grace spectrometer will usually focus on the energy range below the Mn *L*-edge. The second spectrometer is a newly developed plane-grating spectrometer (PGS) consisting of a collimating parabolic mirror followed by a large 1200 lines mm^−1^ grating (Agåker *et al.*, 2009 ▸). The diffracted light is focused onto an MCP detector by a plane parabolic mirror. This optical scheme yields high throughput and good energy resolution between 27 and 200 eV. Both spectrometers use MCP detectors with delay-line readout for high spatial resolution and low readout noise. Delay-line detectors also allow for synchronization to external pulses, such as the bunch marker signal from the storage ring. For instrumentation protection, both spectrometers can be isolated from the experimental chamber with thin filters and windows.

## 4. Beamline performance at the new MAX IV 1.5 GeV ring   

The photon beam is created by the EPU61 insertion device in the 1.5 GeV storage ring. The photon beam intersects the first mirror of the beamline that forms a collimated beam which is monochromated by a plane-grating monochromator (cPGM). Two gratings with blazed grooves are installed: a 1221 lines mm^−1^ with Au coating and a 250 lines mm^−1^ with Ni coating. The Ni-coated grating is dedicated to measurements where improved flux but modest resolution is needed in the photon energy range 200–600 eV. At the time of the beamline transfer process, the Au grating as well as the mirrors were cleaned from carbon contaminants prior to installation to the beamline with UV-light generated ozone. Nevertheless, after a few years of operation there is a clear dark stripe of carbon visible on some of the optical components. The two first mirrors are water-cooled, with the plane mirror having internal cooling channels and the first mirror cooled from the sides. The cooling method was designed in order to be able to handle the heat load coming from the MAX IV 1.5 GeV ring running at 500 mA. The beamline components were manufactured by FMB Berlin except for the gas absorption cell which was made in-house based on a design from Paul Scherrer Institute (Schmitt, 2013 ▸). A detailed study of the mechanical performance of the monochromator is published elsewhere (Sjöblom *et al.*, 2020 ▸).

The monochromated light is directed to one of the branches (either APXPS or RIXS) by one of the two focusing mirrors, which act also as switching mirrors. Both branches have their own gas absorption cells, exit slits and refocusing mirrors. Further information on the photon source and optics can be found elsewhere (Urpelainen *et al.*, 2017 ▸). The general details of the beamline are summarized in Table 2 ▸.

**Figure 5:**
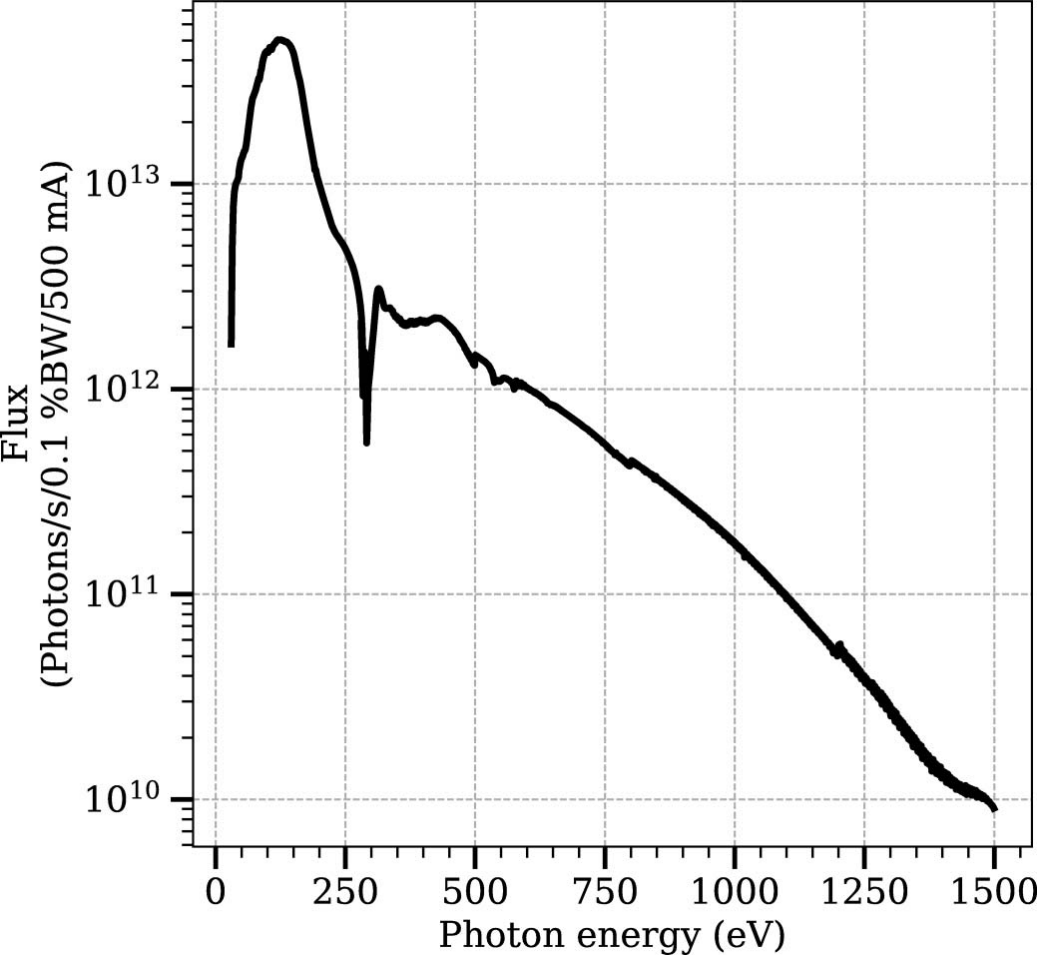
Flux curve measured using a constant photon bandwidth of 0.1%. The measurement is made on a photodiode in the APXPS branch, before the refocusing mirror (M4). The decrease around 280 eV is due to carbon contamination on the optics. Measurement was made with an Au grating using a *c*_ff_ value of 2.25 and with the opening of a beam-defining aperture before the monochromator set to 1 mm × 1 mm. The transmission of the M4 mirror will reduce the flux slightly from these data.

The measured flux curve of the beamline is shown in Fig. 5 ▸. The flux was measured using a photodiode (IRD AXUV100) on the beam position monitor in the APXPS branch, which is located before the final refocusing mirror. During the measurement, the size of the exit slit was varied in each point to reach approximately 0.1% bandwidth. The flux measurement was made using the Au grating with a fixed focus constant (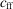) value of 2.25. The flux on the sample position in the APXPS branch is reduced slightly due to the reflection losses in the final refocusing mirror [reflectivity of the Au-coated refocusing mirror (2° grazing incidence angle) varies between approximately 95% and 50% in the photon energy range of the beamline]. When the AP cell is in use, the flux is further reduced due to the absorption in the window material (see Fig. 3 ▸). It is anticipated that cleaning some of the carbon contamination on the optics will give more photon flux above approximately 150 eV. *In situ* cleaning of the optics by leaking in O_2_ gas into the vacuum chambers is expected to start soon. All the vacuum chambers and components therein were designed to comply with a constant oxygen leak, with the Ni grating being an exception since its surface can oxidize upon continuous exposure to O_2_.

### 4.1. Photoabsorption measurements   

The photon energy resolution was measured to show beamline performance at typical settings but also to check the performance with respect to designed values. The resolution was measured using core-level photoabsorption measurement of nitrogen (N_2_) and neon (Ne) gases.

**Figure 6:**
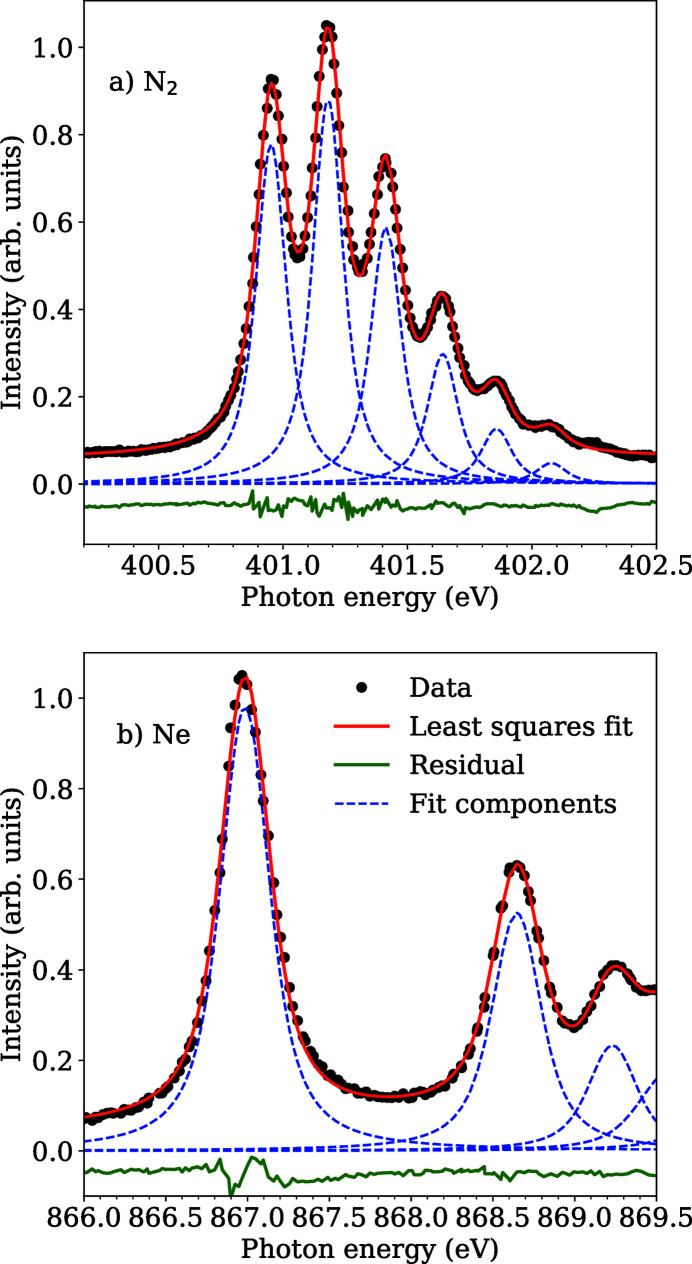
The total ion yield spectra of (*a*) the N1s 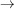 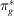 excitation in the N_2_ molecule and (*b*) neon in the Ne 1*s* ^−1^3*p* excitation. The black points indicate measured data points with the fitted curve shown in red with its components as dotted blue lines and the residual as green solid line in both panels. Resolving powers of 6500 and 4500 were reached for N_2_ and Ne cases, respectively.

The N 1*s* photoabsorption spectrum which shows the various vibrational lines from the excitation of the N 1*s* electrons to the 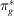 levels in the N_2_ molecule is shown in Fig. 6 ▸(*a*) together with a least-squares fit. In all resolution measurements the Au grating was used with a *c*_ff_ value of 2.25. The spectrum was recorded using a small opening in the entrance of the monochromator (about 0.5 mm × 0.5 mm) and a beamline exit slit opening of 50 µm. A Voigt profile was used for the fit where the Lorentzian width was fixed to be 120 meV (Hitchcock & Brion, 1980 ▸), which gave a Gaussian width of ∼61 meV. At this energy, this gives a resolving power of approximately 6500.

Using Ne gas, the resolution was characterized at higher photon energy as well. Figure 6 ▸(*b*) shows the total ion yield of Ne gas in the photon energy of about 876 eV. The spectrum was recorded with a monochromator entrance opening of 0.5 mm × 0.5 mm and a beamline exit slit opening of 50 µm. The spectrum was fitted with a Voigt profile with fixed Lorentzian width of 254 meV (Coreno *et al.*, 1999 ▸), which resulted in a Gaussian width of 191 meV, giving a resolving power of approximately 4500. The resolution values at both edges are in good correspondence with the expected performance at these settings.

### 4.2. Beam profile measurements   

**Figure 7:**
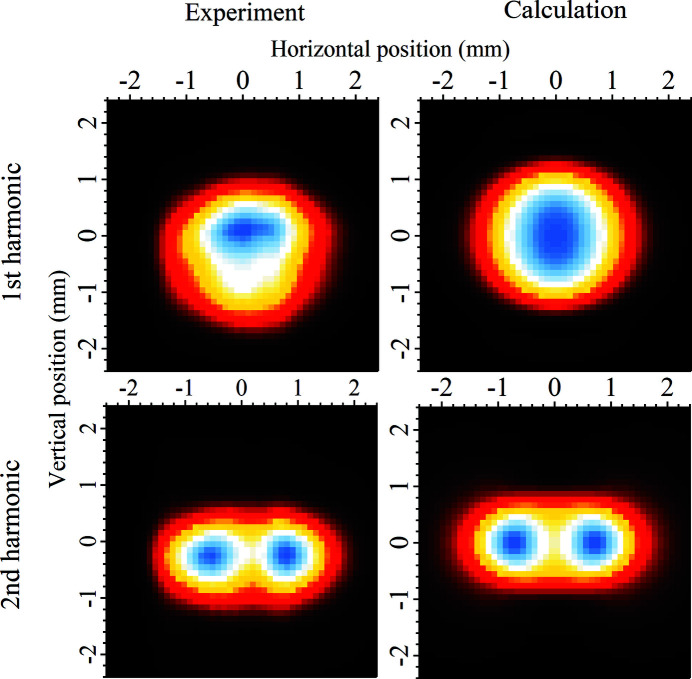
Measured and calculated beam profile maps of the first and second harmonics. The measurement was made using the monochromator baffles placed about 1 m upstream from it. The undulator was tuned to give first harmonic at 50 eV photon energy, and the second harmonic was recorded at 100 eV. The theoretical maps were calculated with the *SPECTRA* software using realistic values for the light source, and the same number of bins/steps as in the experiments.

An essential part of a beamline commissioning work is to ensure that the beam travels through the whole beamline correctly and hits all optical elements in the proper angle and position, resulting in a desired spot at the sample position. This type of commissioning work is often done by observing how the beam appears on various diagnostic elements, such as diodes and fluorescent screens. Another method is to measure undulator spectra to see, among other things, the ratio between even and odd harmonics. During the commissioning work of SPECIES, all of these techniques were used, but we have also characterized the spatial profile of the beam with the use of the baffles situated in front of the monochromator. In these measurements, the baffles were configured to create a rather small opening (in this case 0.5 mm × 0.5 mm) which was then rastered over a specific range. The beam current was subsequently measured on the photodiode which was placed behind the exit slit of the APXPS branch. The results of this measurement are shown in Fig. 7 ▸. The experimental results are compared with the simulated beam profile, which was calculated using the *SPECTRA* software (version 10.2.0) (Tanaka & Kitamura, 2001 ▸).

As can be seen from Fig. 7 ▸, the correspondence between the experiment and theory is good, indicating that the beam goes through the beamline in a manner that is expected. The first harmonic profile shows some asymmetry in the vertical direction, however, which is most likely due to small alignment errors in the beamline optics. With these types of results, it is very obvious if the beam is severely cut, or enters the optics in an incorrect angle, as the shape of the beam profile is quite sensitive to these effects.

## 5. Example research   

### 5.1. Oxidation states of an industrial SCR catalyst using APXPS   

It has been recognized for a long time that XPS is a powerful tool for the investigation of catalyst samples and reaction mechanisms (Briggs, 1980 ▸; Pijpers & Meier, 1999 ▸). Likewise, it also has been recognized that there exists a pressure gap between UHV experiments and real catalytic conditions, which may limit the use of XPS (Knop-Gericke *et al.*, 2009 ▸; Knudsen *et al.*, 2016 ▸) and other surface science techniques (Ertl, 1990 ▸; Lee *et al.*, 1986 ▸) in the study of catalytic samples. APXPS addresses this gap by allowing XPS investigations at more realistic pressures (Starr *et al.*, 2013 ▸; Salmeron & Schlögl, 2008 ▸; Schnadt *et al.*, 2020 ▸). Besides the pressure gap, another gap exists between typical surface science experiments and catalytic applications: the materials gap, which refers to the higher structural complexity of real catalysts in comparison with the model systems of surface science.

The present research example addresses both gaps. It is concerned with the most common industrially relevant catalysts for the selective catalytic reduction of NO_*x*_ by NH_3_ (NH_3_-SCR) made of 3% V_2_O_5_ supported on anatase-TiO_2_ with an admixture of 5% SiO_2_ (3% V_2_O_5_/TiO_2_–5% SiO_2_). In the SCR reaction the redox properties of vanadium play a major role on the reaction as the vanadium ions in the V^5+^ state serve as an adsorption site for the NH_3_ molecules, which reduces it to V^4+^ in the catalytic reaction cycle. Subsequently, the V^4+^ ions are re-oxidized by the O_2_ gas in the reaction environment (Busca & Zecchina, 1994 ▸; Arnarson *et al.*, 2017 ▸).

The redox properties of an SCR catalyst (provided by Dinex Finland) were studied in UHV conditions and at 1 mbar of air. The samples were prepared by diluting approximately 100 mg of the catalyst sample into 5 ml of ethanol, which was then spin-coated onto a gold foil. XPS was carried out under UHV conditions and at a sample exposure to 1 mbar of air. The Au 4*f*_7/2_ core level from the gold foil was used for energy calibration. A Shirley background or a combination of a linear with a Shirley background was subtracted from the spectra. The spectra were measured with an overall energy resolution of approximately 200 meV. The photon energy was chosen so that electrons had a kinetic energy of about 100 eV.

**Figure 8:**
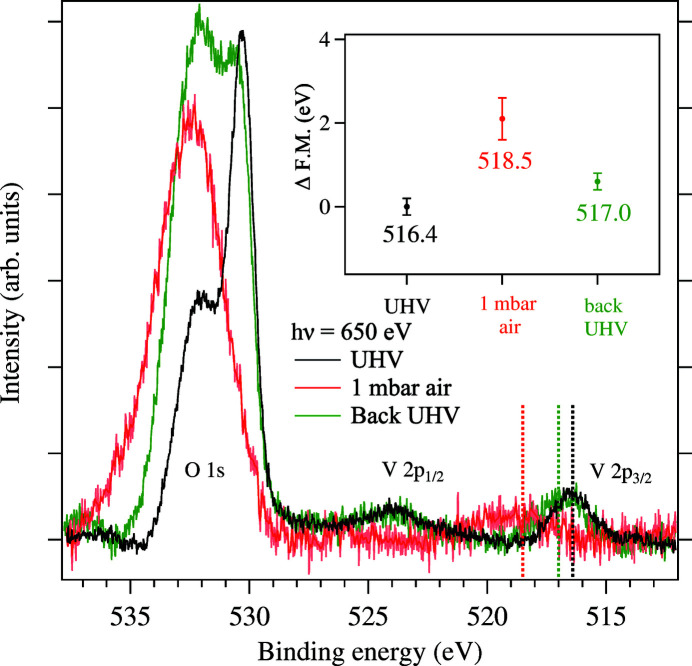
O 1*s* and V 2*p* core-level spectra for the 3% V_2_O_5_/anatase-TiO_2_–5% SiO_2_ catalyst under UHV conditions, during exposure to 1 mbar of air and after evacuation. The spectra are normalized with the V 2*p*_3/2_ area. The inset shows the shift of about 2 eV when exposed to air in the first momentum of the V 2*p*_3/2_ core level.

Exposure of the sample to 1 mbar of air leads to a blueshift of the V 2*p* peak to an energy that is characteristic of the V^5+^ oxidation state (Koust *et al.*, 2018 ▸). Subsequent evacuation to UHV reduces the sample back to the V^4+^ state. The shift in binding energy is highlighted in the inset of Fig. 8 ▸, which shows the statistical first moment of the V 2*p* peaks in the three spectra. In the O 1*s* region, an increase is seen in the binding energy region where hydroxyl groups, adsorbed water and carbon contamination from the air are expected (Sanjinés *et al.*, 1994 ▸; Zimmermann *et al.*, 1998 ▸; Silversmit *et al.*, 2004 ▸). Once the sample is in vacuum again, these components are not removed within the time of this measurement. The finding of a reversible reduction of the vanadium oxide in the catalyst material upon introduction in UHV shows that a UHV environment is not suitable for the study of the SCR catalyst. It emphasizes that pre- and post-analysis methods might not be sufficient to understand chemical reactions and their mechanism on surfaces.

*In situ* and *operando* techniques like Raman spectroscopy, infrared spectroscopy and several other spectroscopic methods (Knop-Gericke *et al.*, 2009 ▸; Busca & Zecchina, 1994 ▸; Topsoe *et al.*, 1995 ▸; Chakrabarti *et al.*, 2017 ▸) have been employed to study the NH_3_-SCR reaction. So far, however, they have not succeeded in drawing a conclusive picture on the reaction mechanism and the active site of the catalytic reaction. We foresee that APXPS might enable us to obtain relevant information about the role and identification of active sites and the reaction mechanism. Experiments with SCR conditions and reactants have been performed. Information from the NH_3_ adsorption and SCR reaction mechanism onto the catalytic surface are expected to be published soon. With information obtained directly from real industrial catalytic materials, we are one step closer to overcoming not only the pressure but also the materials gap and demonstrate that the catalytic industry can benefit from APXPS and synchrotron radiation research.

### 5.2. APXPS of hydrogen on platinum surface   

With this case study we demonstrate the capability of the APXPS endstation to record UPS data at ambient pressures. We have therefore chosen to study the effect of hydrogen adsorption on a platinum surface. Hydrogen is a notoriously difficult element to observe on surfaces with XPS due to its low cross section at typical XPS photon energies and it is thus often said to be impossible to observe (Kerber *et al.*, 1996 ▸; Stojilovic, 2012 ▸). Since the lowest photon energy that SPECIES can produce is 30 eV, it is a suitable beamline to also make measurements on the valence bands levels.

As a sample, we chose a platinum (111) crystal which was initially cleaned with several cycles of Ar^+^ sputtering, oxygen treatments and UHV annealing. The cleanness of the surface was subsequently checked with LEED and XPS.

**Figure 9:**
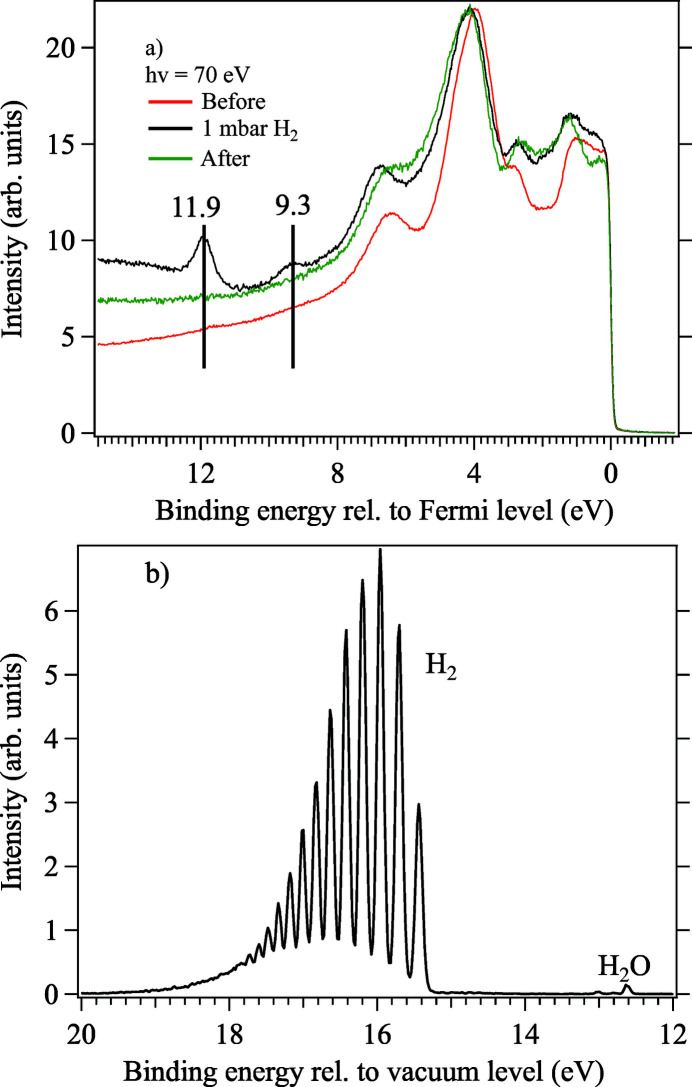
(*a*) Valence band spectra of Pt(111) surface before, during and after exposure to hydrogen gas. The vertical bars indicate positions of the new features that appear upon exposure to H_2_, indicating the presence of H—Pt bonds. (*b*) Valence band spectrum of H_2_ gas phase. Both spectra are measured using a photon energy of 70 eV.

After the initial cleaning, the sample was transferred to the ambient pressure cell and exposed to hydrogen gas at a total pressure of 1 mbar at room temperature. Figure 9 ▸(*a*) shows the valence band measurement before, during and after the H_2_ exposure. The spectra are dominated by the rich *d*-band structure of the Pt surface, but, when the surface is exposed to H_2_, new features appear at binding energies of 11.9 and 9.3 eV on the spectrum as indicated by the vertical lines. These features are at very similar binding energies as those observed by Zhong *et al.* (2018 ▸) and attributed as Pt—H bonds, indicating adsorbed hydrogen. It should be noted that, since our measurement was made at much lower photon energy than that of Zhong *et al.*, we see a much stronger signal due to the higher photoionization cross section.

Figure 9 ▸(*b*) shows a pure gas phase spectrum of the gas introduced to the cell. In this measurement, the Pt sample was retracted out of the way of the synchrotron beam to minimize the secondary electrons that reach the electron analyser. In this spectrum, the vibrational lines from the ionization of the H 1σ_*g*_ valence orbital of the H_2_ molecule are very clearly seen. The pure gas phase spectrum allow us to probe possible impurities introduced into the cell together with the H_2_ gas. In this case, it is clear that some small amounts of water appear in the gas phase as well.

**Figure 10:**
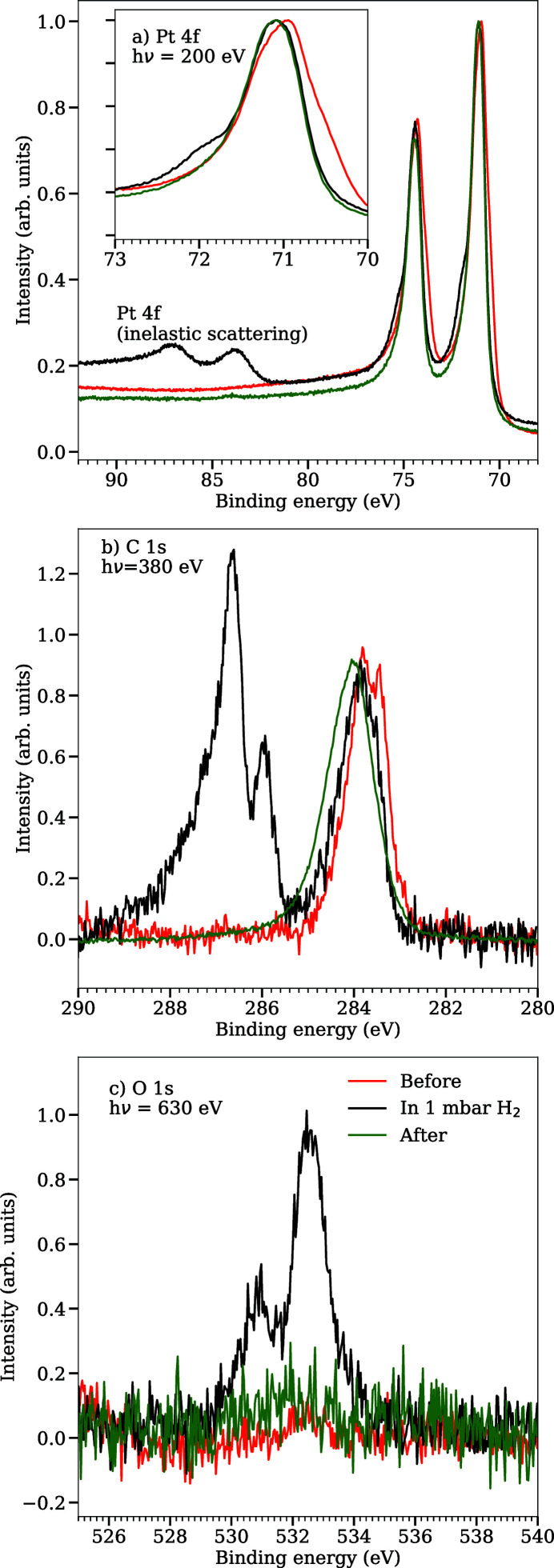
(*a*) Pt 4*f* core-level spectra measured at 200 eV photon energy. The inset shows a close-up view of the Pt 4*f*_7/2_ peak. The spectra are normalized to bulk Pt 4*f* intensity. (*b*) C 1*s* core-level spectra measured at 380 eV photon energy. The C 1*s* spectra are normalized to have equal intensity in the peak at 284 eV. (*c*) O 1*s* core-level spectra measured at 640 eV photon energy. In each core-level, the red spectra are recorded before any H_2_ was added, the blue spectra are recorded when the sample was exposed to 1 mbar H_2_, and the green spectra after the cell had been evacuated.

Figure 10 ▸(*a*) shows the Pt 4*f* core-levels also measured before, during and after H_2_ exposure. The main feature originates from the bulk Pt 4*f* electrons at about 71 eV with surface component observed at about 0.35 eV lower in binding energy. Upon exposure to gas, the surface states decrease to be only a few percent of the bulk and remains low even when the cell is evacuated. During the H_2_ exposure, a new component appears at about 0.8 eV higher binding energy with respect to the bulk peak. The binding energy shift from the surface Pt line to the new component is too large to be attributed to adsorbed hydrogen (Pt—H) bonds. This apparent contradiction between the UPS spectrum that suggest Pt—H bonds and the Pt 4*f*_7/2_ spectrum acquired during exposure that is incompatible with Pt—H bonds indicates that other surface species could be present on the surface.

The O 1*s* and C 1*s* core-levels shown in Figs. 10 ▸(*b*) and 10(*c*) taken at the same time as Pt 4*f* and the valence band indeed gives experimental support for carbon-containing species on the surface during H_2_ exposure. In the C 1*s* spectrum, a peak is observed at 283.8 eV indicating adsorbed carbon. As the sample is exposed to H_2_, two new components arise at binding energies of 285.9 and 286.6 eV fitting well with adsorbed CO in atop and bridge sites, respectively (Björneholm *et al.*, 1994 ▸). Similarly, the O 1*s* spectra display components at 530.9 and 532.6 eV also corresponding to CO in atop and bridge sites.

**Figure 11:**
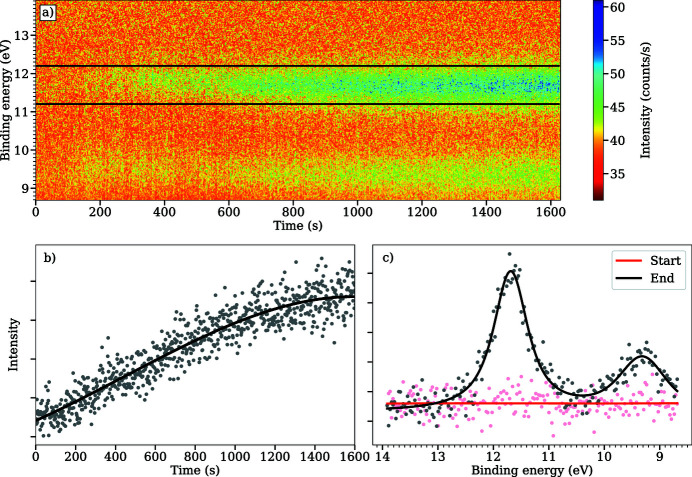
The valence region of the Pt(111) surface as seen in a time-resolved experiment with the hydrogen gas dose. Each spectrum was taken at approximately one-second intervals, thereby the scan number also indicates the amount of time that has passed since the beginning in seconds. The black lines in (*a*) indicate the binding energy region that was used for the integrated signal in (*b*), where the trend of the increase of the signal is shown as a black line. (*c*) The first and last ten spectra of the measurement in red and black, respectively. The spectrum at the end has been fitted with a Voigt curve to show the appearance of the peaks. All measurements were made at a photon energy of 200 eV.

While the sample was initially exposed to the gas, we also recorded time-resolved spectra of the valence band levels using the so-called *snapshot mode* of the analyser. In this mode, the analyser voltages are kept constant and only a fixed kinetic energy range is observed on the detector. As no voltages need to be changed, this mode allows to measure spectra very fast, thereby capturing spectral changes in sub-second timescale. The time-resolved spectra of the valence levels are shown as a colour map in Fig. 11 ▸(*a*), where the *x*-axis denotes the time since the start of the measurement and the *y*-axis the electron binding energy. In the snapshot mode, the energy range of the measurement is determined by the analyser’s pass energy, which in this case was 50 eV, giving a energy window of approximately 5 eV. In this case the energy window was placed so that the features at binding energies of 11.9 and 9.3 eV would fit into the same window. The time-resolved colour map very clearly shows how these two peaks grow as a function of time from the background signal. The intensity of peak at 11.9 eV is additionally integrated in Fig. 11 ▸(*b*) with a fitted trend line indicating the rise in signal intensity. For additional clarify, the sum of the first and last ten spectra from the time-resolved measurement are shown in Fig. 11 ▸(*c*), with fitted Voigt shapes for the two peaks. The time-resolved data were recorded at a photon energy of 200 eV, which is different to what was used to record the spectra in Fig. 9 ▸. Higher photon energy was chosen for the time-resolved measurements in order to obtain higher kinetic energy electrons. In this case, measuring at higher kinetic energy simplifies the background subtraction process considerably, as a linear background can be assumed.

The time-resolved measurement gives additional evidence for CO adsorption on the surface. In this experiment, based on the data in Fig. 11 ▸(*b*), it took approximately 22 min to reach saturation for the peak at 11.9 eV. This is a very long time for the sample to be in 1 mbar of H_2_ and, if the peak would correspond to adsorbed hydrogen, it would appear much faster. A more likely explanation is that small traces of CO in the H_2_ gas, with time, leads to adsorbed CO molecules in the atop and bridge sites. We can also identify the new peaks in the valence band: the peak at 11.9 eV would seem to correspond quite well with the 4σ orbital of CO and the peak at 9.3 eV fits with the mixture of 5σ and 1π levels (Alnot *et al.*, 1982 ▸).

We thus have to conclude that our H_2_ experiment suffers from CO contamination, which is a well known problem in reducing or H_2_ conditions in the APXPS community. While on one hand the results are not what we expected, on the other hand the example underlines one of the strong and rather unique capabilities of the SPECIES beam time: the ability to correlate UV photoelectron spectra with X-ray photoelectron spectra in mbar gas environment. This is, for example, very important for correct interpretation of AP-UPS spectra, which we demonstrate with our example. In fact, correct interpretation of UPS spectra and identification of UV fingerprint signal at mbar conditions is becoming more and more important in the coming years as very powerful laser sources and laboratory UV-sources start to become available for ambient pressure applications.

### 5.3. Silicon wafer RIXS   

**Figure 12:**
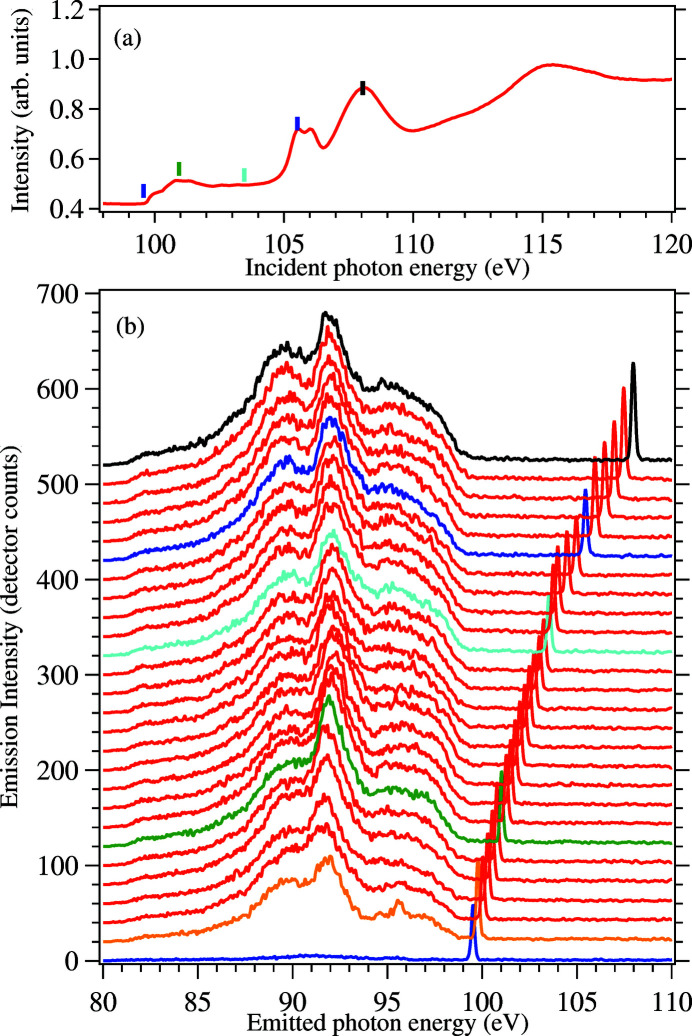
(*a*) Si *L*_2,3_ X-ray absorption spectrum of Si(001) wafer measured in the TEY mode. The coloured vertical lines indicate few selected photon energies corresponding to RIXS measurements. (*b*) Incident photon energy dependent RIXS spectra of Si(001) wafer. From bottom to top, the spectra were measured with *E*_in_ = 99.5 to 108 eV, the steps were set to 0.25 eV between *E*_in_ = 95.5 and 104 eV and to 0.5 eV above *E*_in_ = 104 eV. The black, blue, cyan, green, orange and blue curves represent photon energies depicted with vertical bars of the same colour in (*a*).

To characterize the performance of the RIXS endstation, we chose to investigate the absorption and emission properties of silicon wafer around the Si *L*_2,3_ edge. Figure 12 ▸(*a*) shows the X-ray absorption spectra of Si(001) wafer measured in the RIXS endstation using the total electron yield (TEY) detector. The results agree well with those observed for crystalline silicon (Terekhov *et al.*, 2008 ▸).

Figure 12 ▸(*b*) shows the energy-dependent RIXS spectra of Si wafer with excitation energies scanned through the Si *L*_2,3_ edges. To reduce the strong signal from the elastically scattered photons, the polarization of the incident beam was kept in the scattering plane (π polarization). The entrance slits were set to 80 µm opening and the beamline resolution was set to about 80 meV with the total energy resolution determined from the elastic peak as 200 meV (full width at half-maximum value). All spectra were recorded at room temperature using the Grace spectrometer with the 300 lines mm^−1^ grating. Each spectrum was recorded for 15 min. The top spectrum (black curve) was recorded with incident photon energy of 108 eV which is far above the ionization threshold, reflecting the full partial densities of state of Si. Our results are consistent with the bulk Si results (Kasrai *et al.*, 1993 ▸; Hu *et al.*, 2004 ▸; Terekhov *et al.*, 2008 ▸; Šiller *et al.*, 2009 ▸). At an incident energy of 99.75 eV (orange curve) a sharp feature was observed at 95.7 eV emission energy. This strong resonance implies the transition is due to the valence excitations during the RIXS process. This has been previously theoretically suggested (Minami & Nasu, 1998 ▸; Shirley *et al.*, 2001 ▸).

## 6. Conclusions   

The SPECIES beamline has two branches, with the first one providing a facility for photoelectron spectroscopy in ambient pressures and UHV conditions. The second branch is dedicated to resonant inelastic scattering experiments. These complementary techniques provide a unique place for conducting experiments to study the electronic structure of matter in various depths and at different ambient pressure ranges. The low photon energies accessible at the beamline are demonstrated in this paper to provide information that often is not considered, especially in the APXPS community. The industrial catalyst example is aimed at highlighting the importance of APXPS for the industry and how it is possible to develop and improve our current knowledge from real-world systems. The results from the beamline performance show that it meets the design parameters. Both branches of the beamline are currently accepting users.

## Figures and Tables

**Table 1:** Summary of the different cell parameters Maximum pressures are dictated by the use of windows, which are the same in every cell. Maximum temperatures are limited due to the close proximity of the heater with various O-rings in the cell.

Parameter	Standard	ALD	Sulfur
Maximum pressure	20 mbar	20 mbar	20 mbar
Minimum pressure	10^−6^ mbar	10^−6^ mbar	10^−6^ mbar
Maximum temperature	600°C	∼400°C	600°C
Minimum temperature	−30°C	RT	RT
Windows	Si_3_N_4_ or Al (200 nm)		
Gas inlet setup	Double cone	Dedicated lines	Double cone
QMS probe	Inlet and/or outlet	Outlet	Inlet and/or outlet

**Table 2:** Details of the beamline optics The beam size is given as full width at half-maximum value. The flux value is based on the measurement shown in Fig. 5 ▸ and as discussed in the text. The flux in the cell is an approximation and depends on many factors such as the window material on the cell.

Beamline name	SPECIES
Source	EPU61
Mirrors	Au-coated
Monochromator	cPGM
Energy range (eV)	30–1500
Wavelength range (Å)	413–8.3
Beam size (µm)	
RIXS	5 × 25
APXPS	100 × 100
Flux (photons s^−1^)	5 × 10^13^ to 1 × 10^10^
in AP cell (approximately)	3 × 10^13^ to 5 × 10^9^
